# Compressive stress drives adhesion-dependent unjamming transitions in breast cancer cell migration

**DOI:** 10.3389/fcell.2022.933042

**Published:** 2022-10-04

**Authors:** Grace Cai, Anh Nguyen, Yashar Bashirzadeh, Shan-Shan Lin, Dapeng Bi, Allen P. Liu

**Affiliations:** ^1^ Applied Physics Program, University of Michigan, Ann Arbor, MI, United States; ^2^ Department of Physics, Northeastern University, Boston, MA, United States; ^3^ Department of Mechanical Engineering, University of Michigan, Ann Arbor, MI, United States; ^4^ Department of Biomedical Engineering, University of Michigan, Ann Arbor, MI, United States; ^5^ Department of Biophysics, University of Michigan, Ann Arbor, MI, United States; ^6^ Cellular and Molecular Biology Program, University of Michigan, Ann Arbor, MI, United States

**Keywords:** collective migration, unjamming transition, cell shape, cell–cell adhesion, traction force microscopy

## Abstract

Cellular unjamming is the collective fluidization of cell motion and has been linked to many biological processes, including development, wound repair, and tumor growth. In tumor growth, the uncontrolled proliferation of cancer cells in a confined space generates mechanical compressive stress. However, because multiple cellular and molecular mechanisms may be operating simultaneously, the role of compressive stress in unjamming transitions during cancer progression remains unknown. Here, we investigate which mechanism dominates in a dense, mechanically stressed monolayer. We find that long-term mechanical compression triggers cell arrest in benign epithelial cells and enhances cancer cell migration in transitions correlated with cell shape, leading us to examine the contributions of cell–cell adhesion and substrate traction in unjamming transitions. We show that cadherin-mediated cell–cell adhesion regulates differential cellular responses to compressive stress and is an important driver of unjamming in stressed monolayers. Importantly, compressive stress does not induce the epithelial–mesenchymal transition in unjammed cells. Furthermore, traction force microscopy reveals the attenuation of traction stresses in compressed cells within the bulk monolayer regardless of cell type and motility. As traction within the bulk monolayer decreases with compressive pressure, cancer cells at the leading edge of the cell layer exhibit sustained traction under compression. Together, strengthened intercellular adhesion and attenuation of traction forces within the bulk cell sheet under compression lead to fluidization of the cell layer and may impact collective cell motion in tumor development and breast cancer progression.

## Introduction

Cellular jamming impacts many fundamental biological and disease processes, including embryogenesis (Alert and Trepat, 2020), tissue repair (Nnetu et al., 2012; Ajeti et al., 2019), and tumor growth (Wang et al., 2020; Gensbittel et al., 2021). A jamming transition is a transition from a solid-like state to a fluid-like state in which cellular rearrangements are diminished. Jamming transitions during embryogenesis are typically governed by cell density (Blauth et al., 2021). As a monolayer ages, cells proliferate, slow down, and become jammed as a dense cell layer. Jammed cells are observed to be confined in “cages” of the size of a single cell by their neighbors (Garcia et al., 2015), and the friction between cells increases, leading to reduced collective and individual motion. A dense cell layer in a solid-like state can quickly revert to a flowing state when a wound is inflicted (Chepizhko et al., 2018). During wound repair, the ability of cells to rearrange is essential for closing gaps in epithelial tissues and may be regulated by jamming transitions. Although jamming plays a crucial role in many biological events, the main parameters of cellular jamming remain poorly understood.

Recent research suggests cellular unjamming is involved in tumor growth and cancer progression (Haeger et al., 2014; Staneva et al., 2019; Grosser et al., 2021). During tumor growth, cancer cells proliferate in a dense and confined environment, subjecting tumor cells to solid compressive stress (Northcott et al., 2018). Solid stresses affect tumor pathophysiology by directly compressing cancer and stromal cells and indirectly deforming blood and lymphatic vessels (Jain, Martin, and Stylianopoulos, 2014). For tumors to grow and proliferate, cancer cells must be able to divide and move, and cells in parts of the tumor can fluidize and migrate collectively in an unjamming transition^4^. Here, we investigate the idea that unjamming transitions in cancer cells are driven by compressive stress. As solid stress increases within a tumor, microenvironmental factors may prime cells toward invasive phenotypes, giving rise to cellular rearrangements and enhanced migration (Friedl and Alexander, 2011; Bi et al., 2014).

Cellular rearrangements cease when the cell shape index approaches a critical value (Park et al., 2016). Based on the vertex model, which defines a shape index ( $cell\;perimeter/\sqrt{projected\;cell\;area}$ ) of 3.81 as the jamming threshold (Park et al., 2015), structural rearrangement requires cell shape changes. In this way, cells can overcome the jamming constraints of density by adapting their shapes. Densely packed cells can still move if they elongate (i.e., increase the shape index above 3.81), whereas the tissue becomes jammed as the shape index decreases and approaches the critical value. Thus, the jamming transition can be controlled by the preferred cell shape.

It is known that cell–cell and cell–substrate adhesion forces act together to generate a preferred cell shape (Farhadifar et al., 2007; Bi et al., 2016; Trepat and Sahai, 2018). Adhesions are major sites of force transmission in cells and generally strengthen as cells approach jamming (Garcia et al., 2015). Cell–cell adhesion is mediated by cadherins that are anchored to the cytoskeleton (Charras and Yap, 2018), whereas integrin-dependent cell–substrate adhesion is governed by focal adhesions that generate internal cytoskeletal tension (Kanchanawong et al., 2010). In nonconfluent tissues, decreasing cell–cell adhesion reduces cell crowding and cell–cell contacts, increasing the fluidity of the tissue (Lawson-Keister and Manning, 2021). In confluent tissues, the role of cell–cell adhesion tends to be cell-type specific and depends on the invasive potential of cells (Tse et al., 2012). Strong cell–substrate adhesion combined with high traction stresses is shown to contribute to unjamming in confluent systems (Malinverno et al., 2017; Saraswathibhatla and Notbohm, 2020). Relatively small changes in cell–cell and cell–substrate adhesion can have profound effects on tissue rheology and can be used to regulate cell arrest (Park et al., 2016; Rens and Merks, 2020). How cell–cell and cell–substrate adhesion manipulate jamming transitions in a mechanically stressed monolayer remains unclear.

Here, we characterize the role adhesion complexes play in regulating cellular jamming–unjamming transitions in dense monolayers subjected to mechanical compression. As compressive stress is applied to normal breast epithelial cells (MCF10A) and metastatic breast cancer cells (4T1), MCF10A cells are rendered immobile, while 4T1 cells become fluid-like and migrate as a highly coordinated collective. In the process, 4T1s become elongated and develop strong cell–cell adhesions, whereas E-cadherin is disrupted at the cell–cell contacts of MCF10As. Since mesenchymal markers are not upregulated in compressed 4T1s, this compression-induced transition is distinct from the epithelial-to-mesenchymal transition (EMT). Compression reduces traction stresses in micropatterned cell islands and within the bulk monolayer for both MCF10A and 4T1 cells. Together, we show that upregulated intercellular adhesion and reduced traction within the bulk cell sheet regulate the observed differential cellular responses to mechanical compression and contribute to jamming–unjamming transitions.

## Results

### Long-term compressive stress drives cellular jamming–unjamming transitions

**FIGURE 1:**
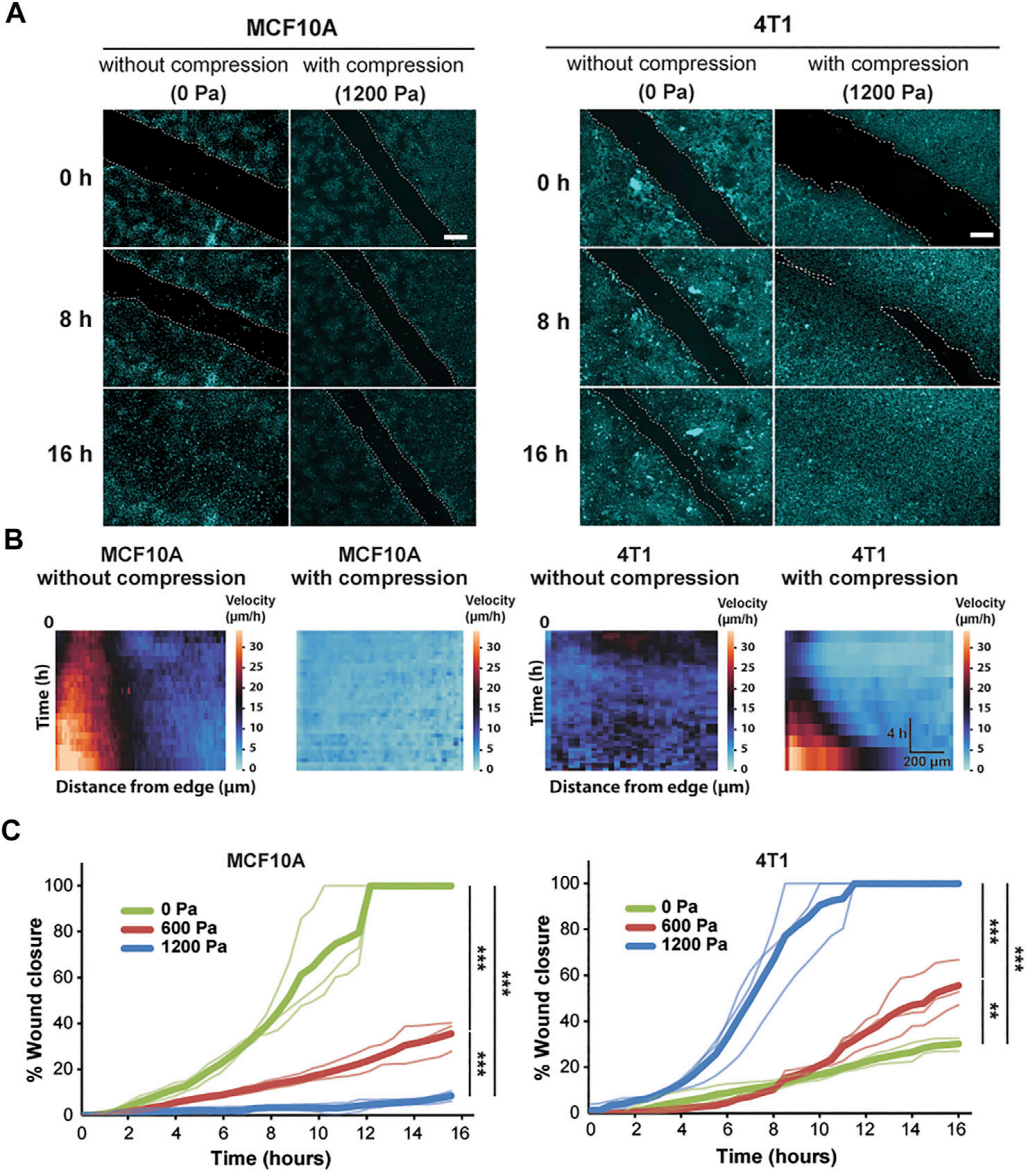
Compressive stress inhibits migration in MCF10A cells and enhances migration in 4T1 cells. **(A)** Representative fluorescence images of MCF10A and 4T1 wound areas at the indicated time points post-wound with and without compression. Cell nuclei are labeled with Hoechst 33342. Lines are scratched on each well using a p-200 pipette tip and cell migration is captured by fluorescence microscopy at 30-min time intervals for 16 h post-wound. Cell edges used to calculate wound area are outlined by white dashed lines. Scale bars, 200 µm. **(B)** Heat maps show spatiotemporal evolution of the velocity for control and compressed cells. **(C)** Quantification of wound area (between white dashed cell edges) for each cell type and compressive pressure. Mean wound area at each time point is plotted from three independent replicates with the individual experiments plotted as thin lines.

We first asked whether the effects of long-term mechanical compression on collective cell migration depended on the invasive potential of cells. Solid compressive stress ranges from 0.1 to 10 kPa have been reported in human tumors and 0.25–8 kPa in murine tumors (Purkayastha et al., 2021). To address this, we conducted wound healing assays of non-tumorigenic (MCF10A) and cancer (4T1) breast epithelial cells subjected to different levels of compressive stress. The 4T1 cell line is a widely used breast cancer model with the capacity to metastasize efficiently to sites affected in human breast cancer and is, therefore, particularly useful for stage IV human breast cancer research (Tao et al., 2008; Yang et al., 2012; Schrörs et al., 2020). Confluent MCF10A and 4T1 monolayers were scratched to create a uniform wound, inducing migration, and normal compressive force was applied to the cells while the wound margin was monitored over 16 h (Figure 1A). This *in vitro* model has been used previously by us and others and mimics the solid compressive stress experienced by cells during tumor development (Tse et al., 2012; Luo et al., 2022).

During wound healing, control MCF10A cells migrated together at a constant velocity to close the wound (Supplementary Video S1). The epithelial sheet was in a seemingly motile but locally jammed state as the cells migrated collectively in a coordinated manner. However, only cells near the wound edge exhibited high cell velocity, suggesting that the cells in the bulk of the monolayer were caged by their neighbors (Figure 1B). Control 4T1 cells displayed low cell velocity, leading to minimal collective migration and failure to close the wound (Figure 1B and Supplementary Video S2). Applying compressive stress to MCF10A cells resulted in exceptionally low migratory activity, and the cells became nearly immobile (Figure 1B and Supplementary Video S3). However, compressed 4T1 cells underwent highly collective, fluid-like migration and abruptly closed the wound (Figure 1A and Supplementary Video S4). By applying different levels of mechanical compression and tracking the wound margin over time, we demonstrated that compressive stress attenuated cell motility in MCF10A cells, which quickly entered a jammed state. On the other hand, 4T1 cells reacted actively to compressive stress by transitioning to a fluid-like state (Figure 1C), and the wound closure rate was positively correlated with the level of external stress applied. The percentage of wound closure after 16 h was not correlated with the initial wound width for all compressive stresses tested (Supplementary Figure S1).

### Unjamming is linked to changes in cell shape and nuclear shape

**FIGURE 2:**
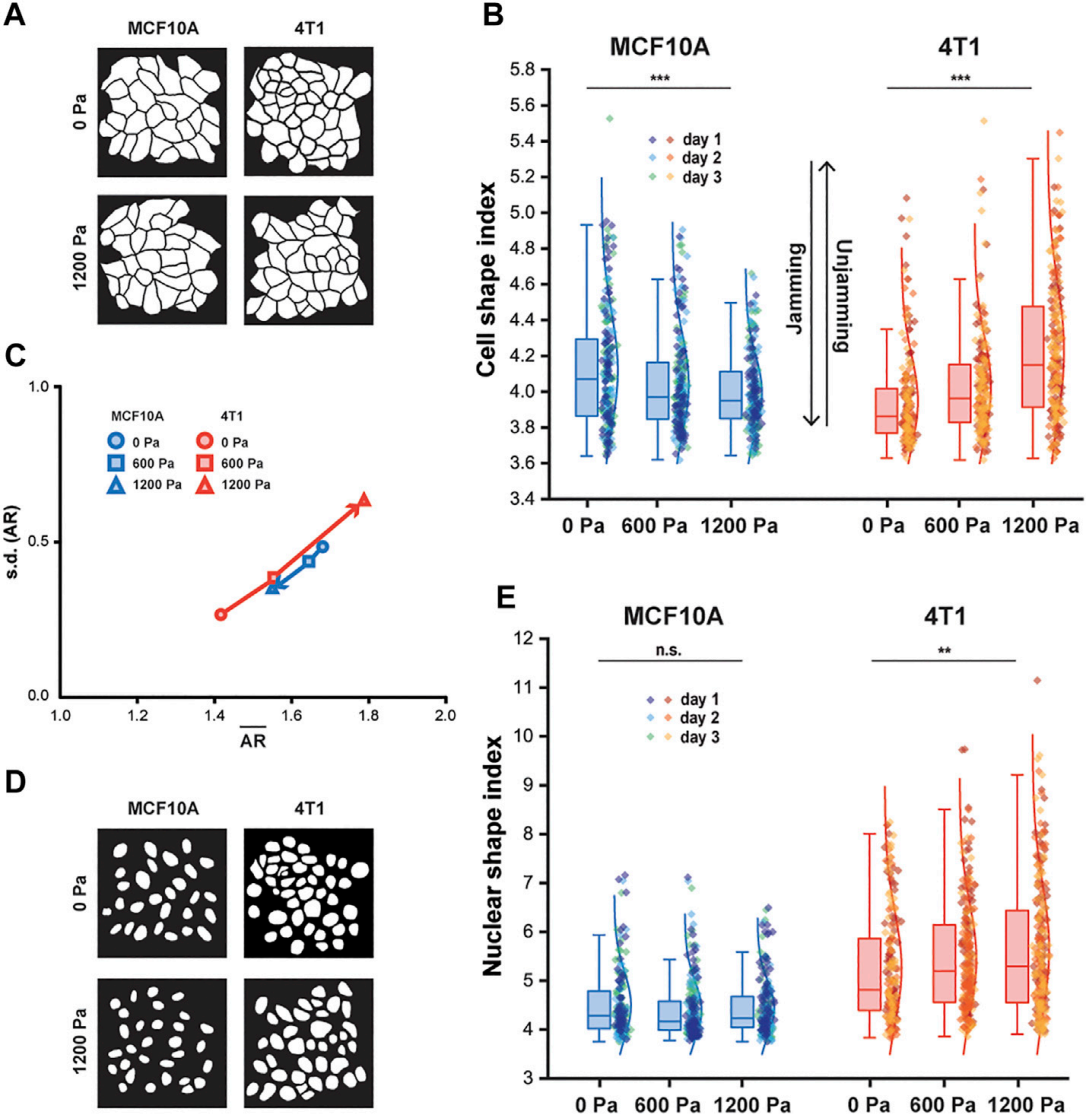
With compression, MCF10A cells and nuclei become more compact, whereas 4T1 cells and nuclei elongate. **(A)** Representative binary images outlining fixed MCF10A and 4T1 cells labeled with E-cadherin with and without compression. **(B)** Boxplot of cell shape index for MCF10A and 4T1 cells subjected to 0, 600, or 1,200 Pa for 12 h. **(C)** Cell aspect ratio (AR), which emphasizes elongation, of control and compressed MCF10A and 4T1 cells is plotted as mean of AR ( $\bar{AR}$ ) vs. standard deviation (s.d.) of AR for each cell type and compressive pressure. **(D)** Representative binary images outlining fixed MCF10A and 4T1 cell nuclei (labeled with DAPI) with and without compression. **(E)** Boxplot of nuclear shape index for control and compressed MCF10A and 4T1 cells. Boxplots of cell and nuclear shape indices show median and quartiles for three independent replicates. Whiskers are maximum and minimum data points, and data from each replicate is denoted as a different color. Number of MCF10A cells and nuclei analyzed: 0 Pa (*n* = 234), 600 Pa (*n* = 229), and 1,200 Pa (*n* = 222). Number of 4T1 cells and nuclei analyzed: 0 Pa (*n* = 205), 600 Pa (*n* = 231), and 1200 Pa (*n* = 224).

We next investigated cell shape as a marker for cellular fluidity in mechanically stressed monolayers. Previously published work used the vertex model to define a critical shape index of 3.81 as the jamming threshold (Bi et al., 2016). Studies using human bronchial epithelial cells show that regardless of the magnitude of intracellular stress fluctuations, the cellular rearrangements cease when the shape index approaches the jamming threshold (Park et al., 2015). Applying compressive stress to the MCF10A cells lowered the shape index, which approached the critical value of 3.81 (Figures 2A,B; Supplementary Figure S2), leading to more compact cells. In contrast, the cell and nuclear shapes of the compressed 4T1 cells became elongated (higher shape index) and more variable compared to those of the control cells (Figure 2B). Although the shape index depends on elongation and tortuosity, the cell aspect ratio (AR) emphasizes elongation and deemphasizes tortuosity (Mitchel et al., 2020). In 4T1 cells, substantial increases in AR were accompanied by smaller increases in shape index (Supplementary Figure S3A), resulting in elongated cells with straight edges. Therefore, cell elongation can be better captured by plotting the mean of AR vs. the standard deviation (s.d.) of AR. We found this to have a positive linear relationship (Figure 2C), in agreement with what has been shown previously (Atia et al., 2018). As the cell AR increased with compressive stress, indicative of unjamming, its variability from cell to cell increased. Compressed cells tended to have higher values of $\bar{AR}$ and s.d. of AR. Increased cell elongation and shape variability suggest more disordered cell packing and fluid-like behavior and may be an indicator of increased metastatic potential. These results, together with the earlier wound healing observations, demonstrate a correlation between cell shape and jamming–unjamming transitions.

Along with cell shape, the nuclear shape has recently been linked to tissue fluidity (Grosser et al., 2021) and is a critical marker for tumor aggressiveness in clinical cancer grading (Denais and Lammerding, 2014). Cancer cell nuclei are generally larger and softer than non-malignant cell nuclei (Fischer, Hayn, and Mierke, 2020; Rianna, Radmacher, and Kumar, 2020; Gensbittel et al., 2021), and studies of multiple cancer cell types, including breast cancer cells, have found that the cells and their nuclei become significantly softer upon extravasation (Roberts et al., 2021). Since nucleus deformability is known to play a central role in cell motility in dense environments (Friedl, Wolf and Lammerding, 2011), we next asked whether changes in nuclear shape were correlated with changes in cell shape in cells subjected to mechanical compression. As 4T1 cells elongated with compression, a higher cell shape index corresponded to an increased nuclear shape index and high variance in nuclear shape (Figure 2E), which has been associated with more aggressive tumors (Grosser et al., 2021). Our results show that cell and nuclear shape indices increase with compressive stress in unjamming transitions and are important indicators of cell motility and tissue fluidity. More importantly, mechanical compression resulted in elongated cell and nuclear shapes in metastatic 4T1 cells, which became unjammed, but not in non-tumorigenic, jammed MCF10A cells.

### The compression-induced unjamming transition is distinct from EMT

**FIGURE 3:**
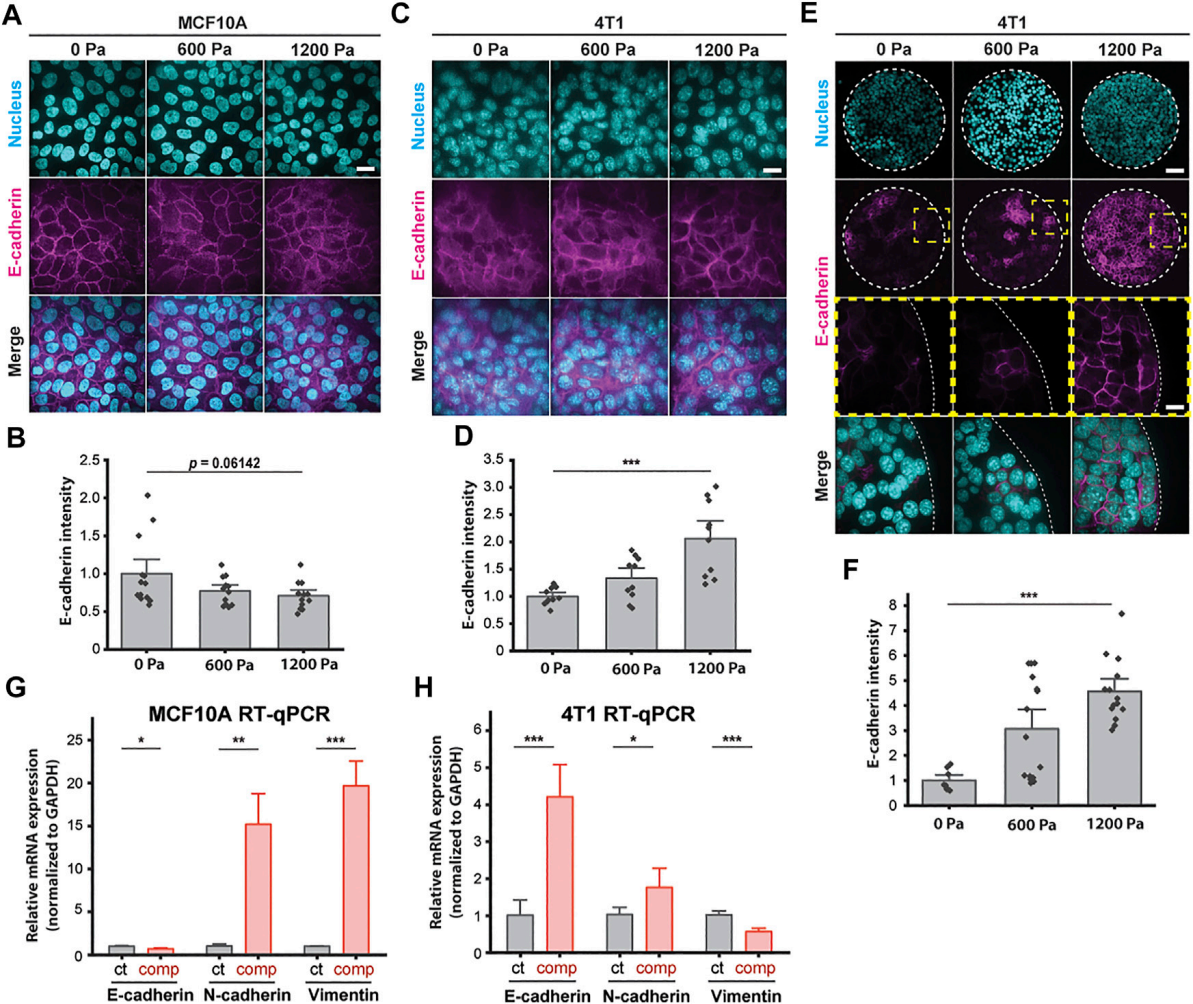
E-cadherin is upregulated in unjammed 4T1 cells under mechanical compression. **(A)** Representative immunofluorescence images of MCF10A cells labeled with DAPI and an E-cadherin antibody. Cell monolayers are subjected to specified compressive pressures for 12 h. Scale bar, 20 µm. **(B)** Quantification of relative E-cadherin fluorescence at MCF10A cell–cell contacts. Mean fluorescence intensity at the cell membrane ±S.E. is plotted from three independent replicates (*n* = 12–13). **(C)** Representative microscopy images of 4T1 cells labeled with DAPI and an E-cadherin antibody in the same experimental conditions as in **(A)**. Scale bar, 20 µm. **(D)** Quantification of relative E-cadherin fluorescence at 4T1 cell–cell contacts. Mean fluorescence intensity at the cell membrane ±S.E. is plotted from three independent experiments (*n* = 10). **(E)** Representative microscopy images of microcontact-printed 4T1 cell islands labeled with DAPI and an E-cadherin antibody. Micropatterned cell islands are exposed to specified stresses for 12 h. Scale bars, 80 µm (top) and 20 µm (bottom). **(F)** Quantification of relative E-cadherin fluorescence at the cell–cell contacts of 4T1 cell islands. Mean fluorescence intensity at the cell membrane ±S.E. is plotted from three independent replicates (*n* = 8–15). qPCR analysis of E-cadherin, N-cadherin, and vimentin mRNA levels with and without compression (1,200 Pa) for MCF10A **(G)** and 4T1 **(H)** cell monolayers. Transcript levels are calculated using the ΔΔC_t_ method normalized to GAPDH. Mean mRNA level ±S.E. is plotted from three independent experiments with duplicates per experiment.

To further elucidate how compressive stress impacts the cell–cell organization, we next investigated the factors which shape cells and support migration in a mechanically stressed monolayer. We first examined the intensity of E-cadherin at adherens junctions after long-term compression. Immunofluorescence staining revealed that compressive stress disrupted E-cadherin localization at the intercellular contacts of the MCF10A cells (Figures 3A and B). Surprisingly, compressed 4T1 cells gained strong cell–cell contacts evidenced by the increased recruitment of E-cadherin to cell junctions (Figures 3C and D), which was also strongly evident in micropatterned 4T1 cell islands (Figures 3E and F). To form micropatterned cell islands, microcontact printing was used to generate fibronectin-coated, circular adhesive islands with a diameter of 400 μm. Compressed 4T1 cell islands showed higher E-cadherin expression and localization to the cell–cell membrane. In a wound healing assay, the lack of collective migration exhibited by 4T1 cells in the absence of applied stress can be attributed to weak cell–cell adhesion. As compressive stress strengthened intercellular adhesion, strong adhesion forces maintained high cell density and encouraged highly directed and coordinated migration, leading to fluidization of the cell layer. Furthermore, compression-induced changes in E-cadherin expression were correlated with cell shape index, with elongated cells having increased localization of E-cadherin to intercellular contacts (Supplementary Figure S3B). This result supports that adhesion strength is higher in a more unjammed cell layer.

To identify a possible mesenchymal molecular signature in compressed cells that attributes increased migratory capability to EMT (Bi et al., 2016; Mitchel et al., 2020), we conducted qPCR assays to quantify the mRNA levels of epithelial marker E-cadherin and mesenchymal markers N-cadherin and vimentin. Compressive stress significantly downregulated E-cadherin and upregulated vimentin in MCF10A cells (Figure 3G). Surprisingly, E-cadherin was upregulated in compressed 4T1 cells by ∼4-fold, while vimentin was downregulated (Figure 3H)—these features are associated with mesenchymal–epithelial transition (MET), as opposed to EMT. N-cadherin was upregulated by compression in both cell types, although upregulation was higher in MCF10A cells. Altogether, we find that enhanced collective migration and a higher cell shape index are associated with increased levels of cell–cell adhesion (Figure 2 and Figure 3). The upregulation of E-cadherin and downregulation of vimentin in unjammed 4T1 cells suggest that the factors governing unjamming transitions in this system are distinct from the transitions between epithelial and mesenchymal cells.

### Cadherin-mediated cell–cell adhesion is required for unjamming

**FIGURE 4:**
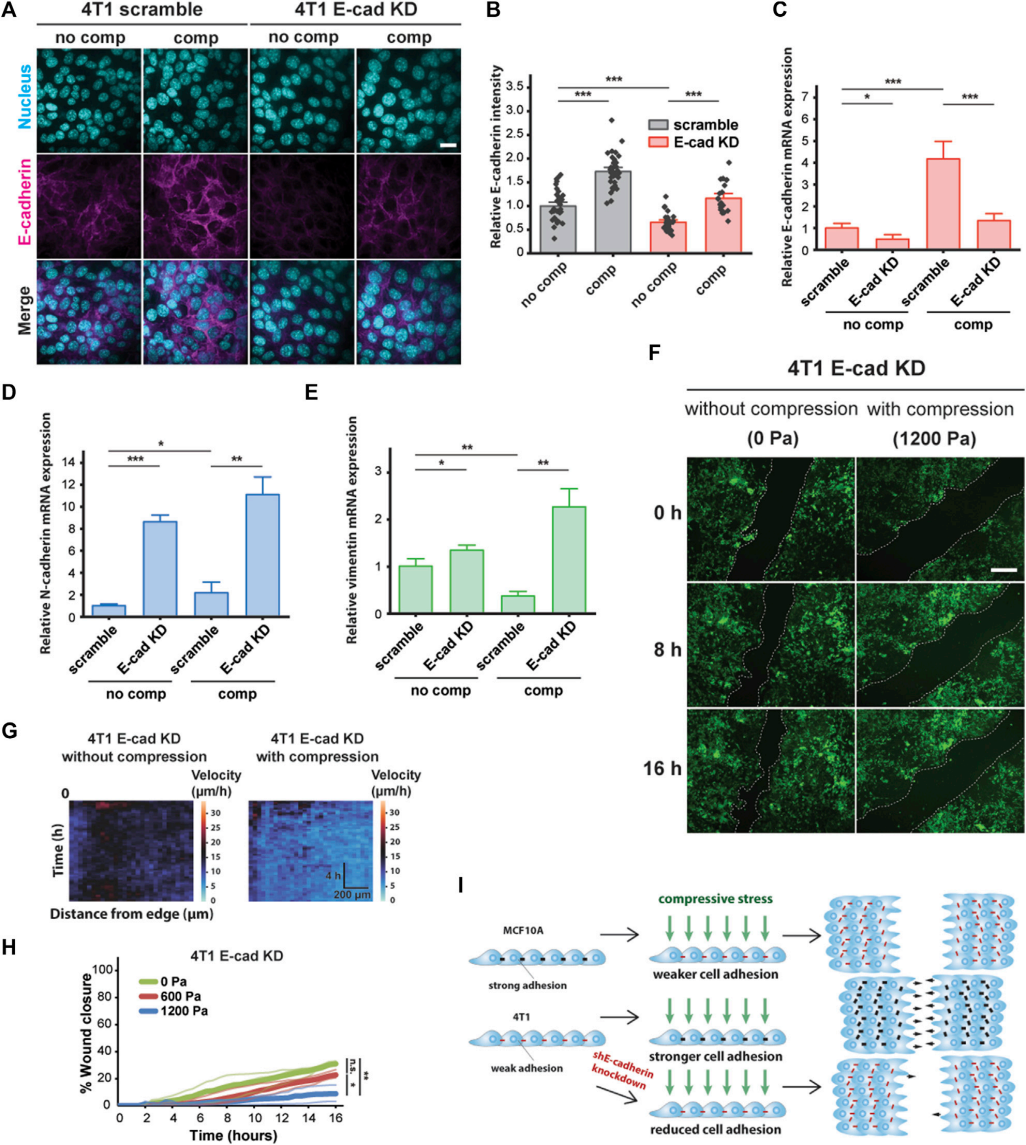
E-cadherin knockdown inhibited compression-induced upregulation of E-cadherin in 4T1 cells, triggering jamming. **(A)** Representative microscopy images of 4T1 scramble and E-cad KD cells labeled with DAPI and an E-cadherin antibody. E-cad KD is induced in 4T1 shE-cadherin cells by adding 200 μM IPTG 72 h prior to experiments. Cell monolayers are exposed to specified stresses for 12 h. Scale bar, 20 µm. **(B)** Quantification of relative E-cadherin fluorescence at the cell–cell contacts of 4T1 scramble and E-cad KD cells. Mean fluorescence intensity at the cell membrane ±S.E. is plotted from three independent replicates (*n* = 22–36). qPCR analysis of E-cadherin **(C)**, N-cadherin **(D)**, and vimentin **(E)** mRNA levels in 4T1 scramble and E-cad KD cells. Transcript levels are calculated using the ΔΔC_t_ method normalized to GAPDH. Mean mRNA level ±S.E. is plotted from three independent experiments with duplicates per experiment. **(F)** Representative images of 4T1 E-cad KD wound area at the indicated time points post-wound. 4T1 shE-cadherin cells express mNeonGreen. Cell edges used to calculate wound area are outlined by white dashed lines. Scale bar, 200 µm. **(G)** Heat maps of spatiotemporal evolution of the velocity for 4T1 E-cad KD cells under different levels of mechanical compression. **(H)** Quantification of wound area (between white dashed cell edges) for each condition. Mean wound area at each time point is plotted from three independent replicates as a representative trace. **(I)** Summary depicting the effect of compressive stress on collective migration in MCF10A WT, 4T1 WT, and 4T1 E-cad KD cells. Strong cell–cell contacts are denoted by black dashes. Red dashes indicate weak cell–cell adhesion. Number of small black arrows (right) represent relative cell velocity during wound healing.

To probe the role of E-cadherin further, we attenuated cell–cell adhesions by knocking down E-cadherin (encoded by CDH1 gene) in 4T1 cells and tested the effect of knockdown on compression-induced unjamming compared to cells transduced with scramble shRNA (Figures 4A and B). Upregulated E-cadherin in unjammed cells suggests a role for cell–cell adhesion in dense monolayers under mechanical compression, and knockdown of E-cadherin is known to switch the migration mode of cells from collective to single-cell migration (Mendonsa et al., 2018). Knocking down E-cadherin initially increased cell motility, consistent with recently published work (Ilina et al., 2020); however, the collective movement was substantially inhibited in E-cadherin knockdown (E-cad KD) cells upon compression (Supplementary Figure S4). Upregulation of E-cadherin expression by compression was significantly reduced in E-cad KD cells relative to scramble cells (Figure 4C). This upregulation was not due to recovery of E-cadherin expression as expression levels remained downregulated over the course of 18 h when uncompressed (Supplementary Figure S5). Additionally, the mesenchymal markers N-cadherin and vimentin were upregulated in KD cells and further upregulated by compression (Figures 4D and E). There is a possibility of inhibiting vimentin in E-cad KD cells to further investigate the potential role of this mesenchymal marker in jamming–unjamming transitions. Applied compressive stresses of 600 and 1,200 Pa decreased the collective migration of E-cad KD cells (Figures 4F and G), lowering the rate of wound closure from ∼31% for 0 Pa to ∼9% for 1,200 Pa (Figure 4H). Our results indicate that E-cad KD cells existed in a jammed state when compressed, similar to what we observed in MCF10A cells.

To assess the generality of our findings, we explored the effect of mechanical compression on the nonmetastatic mouse breast cancer cell line 67NR, which is derived from the same primary breast cancer as 4T1 and expresses N-cadherin and vimentin, but not E-cadherin (Dykxhoorn et al., 2009). Although 67NR cells have been shown to exhibit increased cell motility attributed to higher cell–substrate adhesion under compression (Tse et al., 2012), we did not observe unjamming behavior in compressed 67NR cells (Supplementary Figure S6A), suggesting that expression and localization of E-cadherin are required for compression-induced, fluid-like unjamming transitions. Consistent with our findings for MCF10A, compressive stress did not increase E-cadherin levels in 67NR cells (Supplementary Figure S6B). Based on our findings, schematically summarized in Figure 4I, that compressive stress significantly inhibited the coordinated migration of 4T1 E-cad KD cells and that upregulation of E-cadherin is required for compression-induced unjamming, we conclude that cell–cell adhesion is a key regulator and effector upon compression.

### Compressive stress reduces traction forces within the bulk cell sheet

**FIGURE 5:**
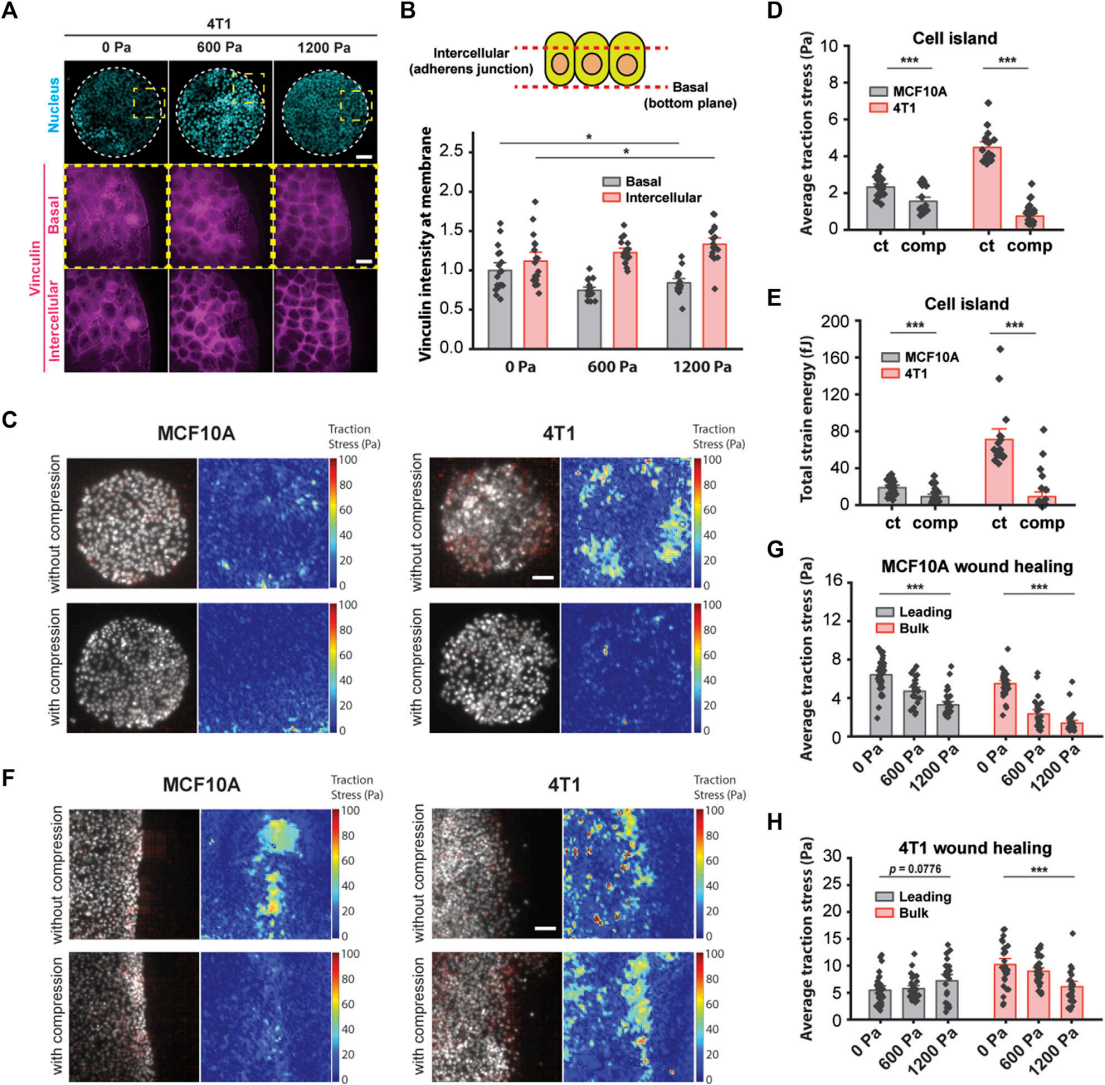
Compressive stress reduced traction stresses within the bulk cell sheet. **(A)** Representative microscopy images of 4T1 cell islands labeled with DAPI and a vinculin antibody. Vinculin staining is shown at two different imaging planes. Micropatterned cell islands were exposed to specified pressures for 12 h. Scale bars, 80 µm (top) and 20 µm (bottom). **(B)** Quantification of relative vinculin intensity of individual cells at the basal plane and at intercellular junctions. Mean fluorescence ±S.E. is plotted from three independent replicates (*n* = 18). **(C)** Traction stress vector field and traction stress magnitude of micropatterned cell islands with and without 1,200 Pa compression for 3 h. Cell nuclei are labeled with Hoechst 33342. Scale bar, 80 µm. Mean traction stresses **(D)** and total strain energy **(E)** with and without compression on micropatterned islands. Number of images analyzed for MCF10A: control (*n* = 21) and compressed (*n* = 21). Number of images analyzed for 4T1: control (n = 18), compressed (*n* = 30). **(F)** Traction stress vector field and traction stress magnitude of a wounded edge with and without 1,200 Pa compression for 3 h. Cell nuclei are labeled with Hoechst 33342. Scale bar, 80 µm. Mean traction stresses exerted by MCF10A cells **(G)** and 4T1 cells **(H)** at the leading edge (within 5–7 cell layers of the wound margin) and within the bulk monolayer for 0, 600, and 1,200 Pa compressive stress for 3 h. Number of images analyzed for MCF10A: 0 Pa (*n* = 37), 600 Pa (*n* = 26), and 1,200 Pa (*n* = 32). Number of images analyzed for 4T1: 0 Pa (*n* = 28), 600 Pa (*n* = 33), and 1,200 Pa (*n* = 24).

We have shown thus far that increased cell–cell adhesion promotes the unjamming of mechanically compressed 4T1 cancer cells. Since high-traction stresses have been shown to reverse the effect of density on shape-dependent cellular rearrangements (Saraswathibhatla and Notbohm, 2020) and to contribute to unjamming in a confluent monolayer (Malinverno et al., 2017), we next explored the role of substrate traction as a potential parameter working together with cell–cell adhesion to promote unjamming in tissues. Vinculin is a cytoskeletal protein responsible for regulating integrin-mediated cell adhesion and is found in focal adhesions as well as adherens junctions (Kanchanawong et al., 2010). In micropatterned 4T1 cell islands, compressive stress reduced vinculin intensity at the basal plane and enhanced the enrichment of vinculin at adherens junctions (Figures 5A and B). A decrease in vinculin intensity at the basal plane is indicative of lowered cell–matrix adhesion, while increased vinculin at the cell–cell membrane is consistent with the increased localization of E-cadherin (Figures 3E and F) since vinculin also binds E-cadherin *via* alpha- and beta-catenin and is needed to form cell–cell contacts. We turned to traction force microscopy (TFM) to further disentangle the individual contributions of cell–cell adhesion and substrate traction (Lawson-Keister and Manning, 2021).

In line with recently published work that showed that the perturbation of intercellular adhesion (by inactivation of the E-cadherin gene) increased traction forces (Balasubramaniam et al., 2021), we used microcontact printing to generate circular adhesive islands with a diameter of 400 μm on a soft silicone substrate coated with fluorescent beads. 4T1 cell islands with low cell–cell adhesion exerted high traction (Figure 5C), and consistent with this, MCF10A cell islands that expressed high intercellular adhesion had low traction stresses (Figure 5C). Applying compressive stress to micropatterned cell islands for 3 h reduced traction for both cell types (Figure 5C). Compressing MCF10A cell islands by 1,200 Pa reduced average traction stresses by 33.6% and strain energy by 52.1% (Figures 5D and E). Compressive stress also largely obliterated traction forces in 4T1 cell islands; average traction forces and total strain energy decreased by 83.5% and 87.1%, respectively, compared to control cell islands (Figures 5D and E). Together with earlier data from Figures 3E and F, compression significantly elevated the cell–cell adhesion in 4T1 cell islands while reducing cell–substrate stresses. Since microcontact-printed cell islands lack the leading edges of coordinated migration, these results suggest that the traction forces of cells within the bulk monolayer are reduced by compressive stress regardless of collective cell motility or the cell type we looked at.

Our observations using micropatterned cell islands indicated that cell–substrate stresses may not be the principal determinant of compression-driven unjamming in breast cancer cell migration. However, considering that traction forces were measured on microcontact-printed cell islands, which do not permit coordinated migration, we cannot completely rule out the contribution of cell–substrate contraction. Previous studies of collective migration during wound healing suggest that the leading edge of a cell sheet presents more cell–substrate adhesion than follower cells (Tse et al., 2012), which enables coordinated migration. We grew monolayers of MCF10A and 4T1 cells on a silicone substrate containing fluorescent beads. We initiated a wound healing assay and then applied mechanical compression to the cells for 3 h. Subsequentially, we observed that traction forces were localized to the leading edge (within 5–7 cell layers of the wound margin) of MCF10A cell sheets and were attenuated by compressive stress (Figure 5F). Average traction stresses at the leading edge of compressed MCF10A cell sheets were reduced by 26.5% (600 Pa) and 48.9% (1,200 Pa) (Figure 5H). Control 4T1 cells generally exerted higher traction throughout the cell layer than MCF10A cells. Compression of 4T1 cell sheets by 1,200 Pa diminished the average traction by 40.3% within the bulk monolayer, while changes in traction at the leading edge were not statistically significant, although traction stresses appeared to be more localized to the leading edge (Figures 5F and H). As traction stresses within the bulk monolayer decreased significantly with compressive stress, cells at the leading edge maintained high traction under compression (Figure 5H). Given these results, our observations point to a differential leader–follower traction force response to compressive stress (i.e., reduced traction within the bulk monolayer and sustained traction at the leading edge) as a contributing factor in the unjamming behavior of 4T1 cells. Altogether, mechanical compression strengthened intercellular adhesion and attenuated traction forces exerted by bulk cells, leading to fluidization of the cell sheet.

### Theoretical simulation using the SPV model suggests distinct paths of jamming–unjamming

**FIGURE 6:**
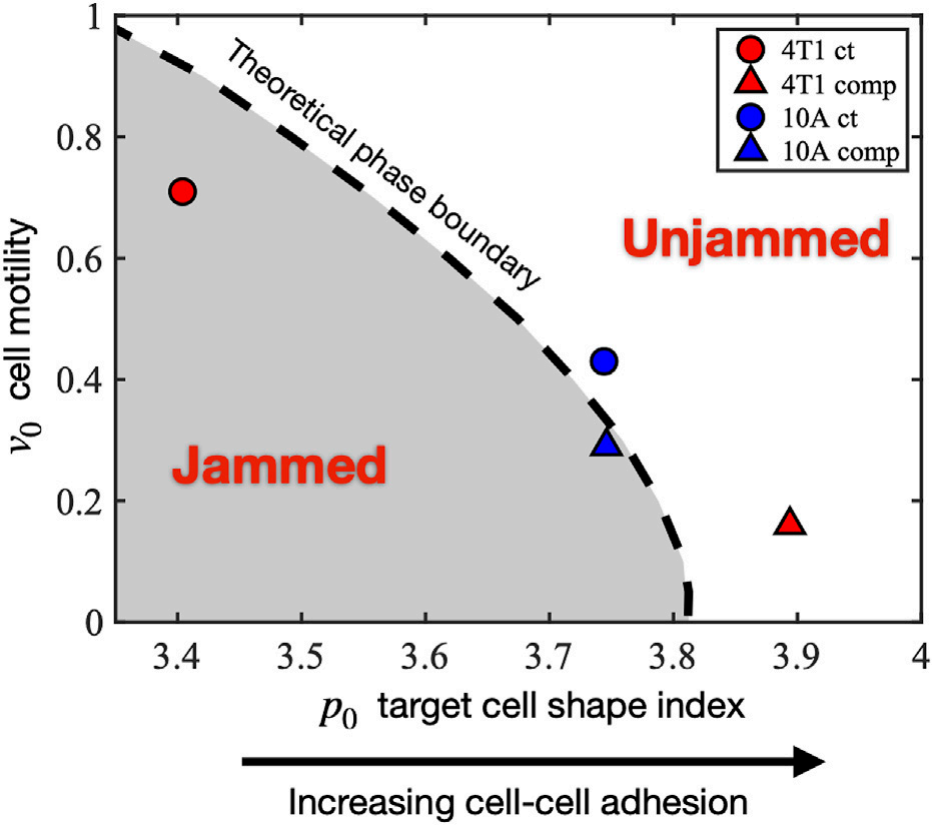
Mapping the experimentally observed tissue states to the theoretical jamming–unjamming phase diagram. The jamming–unjamming phase diagram is shown in terms of the two pertinent parameters of the SPV model: the single-cell motility *v*_0_ and the target cell shape index *p*_0_. By mapping the experimentally observed cell traction forces and cell shapes to theoretical simulation results, the positions of 4T1 and 10A cells are placed on the phase diagram (see Methods).

Our observed experimental data show that there are two distinct responses to long-term compression from the 2 cell types of interest. The initially jammed 4T1 cells become unjammed under compression, whereas the MCF10A cells behave the opposite way, in a cell-adhesion-dependent manner. Seeking a theoretical explanation for this distinction, we investigated the self-propelled Voronoi (SPV) model and mapped the observed cell’s condition to a phase diagram of two model parameters: 1) cell motility $v_{0}$ and 2) target cell shape index $p_{0}$ (the model and parameters are elaborated in more detail in the Method section). The SPV model is well-adapted to capture cell morphological metrics, which are crucial for studying cellular collectives. In the SPV model, the effect of cell–cell adhesion is captured by the parameter target shape index $p_{0}$ (Bi et al., 2015). In this theoretical model, the effects of increased cell–cell adhesion lead to a higher value of $p_{0}$ . From the experimental results measuring E-cadherin fluorescence of MCF10A cells (Figure 3B), which shows an insignificant difference in relative E-cadherin intensity between control MCF10A and compressed MCF10A cells, we expect to see in the phase diagram that the difference in $p_{0}$ of controlled and compressed MCF10A cells is small. This expectation is observed in Figure 6, where $p_{0}$ of the mapped control MCF10A cells is 3.744, while that of the mapped MCF10A compressed cells is 3.746. On the contrary, Figure 3D shows a significant difference in relative E-cadherin fluorescence between control and compressed 4T1 cells. 4T1 cells under long-term compression express much more E-cadherin, which suggests a drastic increase in $p_{0}$ for 4T1 cells under the effect of compression. This feature is also observed in the phase diagram, where $p_{0}$ of 4T1 cells increases from 3.404 to 3.894 in response to compression. This E-cadherin– $p_{0}$ comparison solidifies our experimental–theoretical mapping.

Under the effect of compression, the initially jammed 4T1 cells become unjammed, crossing the phase boundary, and undergoing a decrease in $v_{0}$ and an increase in $p_{0}$ . In the model, this corresponds to decreased single-cell motility and increased cell–cell adhesion. In contrast, MCF10A cells went from unjammed to jammed as a result of compression, suffering a small decrease in $v_{0}$ and no change in $p_{0}$ . Using a jammed–unjammed boundary, we were able to see two distinct transition paths taken by 4T1 and MCF10A cells under mechanical compression.

## Discussion

In the present study, we show that cell–cell adhesion is an important mechanism for unjamming transitions in a mechanically stressed monolayer. We first establish increased collective cell migration in a transition correlated with cell shape and distinct from EMT. Increased migratory behavior has traditionally been associated with EMT (Thiery et al., 2009; Nieto et al., 2016; Jolly et al., 2017), during which cells lose epithelial features and become mesenchymal. However, in the transition we observed, coordinated migration is enhanced, and the tissue fluidizes as the epithelial marker E-cadherin is upregulated and the mesenchymal marker vimentin is downregulated. These findings support a recently published characterization of the unjamming transition in which epithelial character is maintained without gaining mesenchymal character (Mitchel et al., 2020). As cell–cell adhesion increases, cells undergo a fluid-like unjamming transition, during which collective cell motion is increased and wound repair is accelerated. We discover that E-cadherin knockdown inhibits coordinated migration under mechanical compression, demonstrating that strong cell–cell adhesion, accompanied by the accumulation of vinculin at intercellular contacts, is important for regulating unjamming transitions. The attenuation of traction stresses in micropatterned MCF10A and 4T1 cell islands suggests that substrate traction may not be the dominant parameter in compression-induced unjamming transitions. Further investigation of cell traction during wound healing suggests that in a compressed 4T1 cell sheet, the cells within the bulk monolayer present substantially lower traction forces and higher cell–cell adhesion, allowing for greater structural rearrangements and highly correlated, fluid-like migration, where the bulk cells are more easily pulled by the cells at the leading edge. Our results suggest that 4T1 cells respond to compressive stress by elongating, strengthening cell–cell adhesions, and reducing traction within the bulk monolayer, leading to increased fluidization of the cell sheet and highly coordinated migration during wound healing.

One open question is how compressive stress leads to increased recruitment of E-cadherin to promote cell–cell adhesion. Mechanical stimuli are known to remodel epithelial cell–cell junctions by junction elongation and contraction mediated *via* mechanosensitive channels (Varadarajan et al., 2022). It is also well documented that adherens junctions become reinforced when cells are under tension (Borghi et al., 2012; Charras and Yap, 2018; Pinheiro and Bellaïche, 2018). We have previously found that mechanical compression activates mechanosensitive channel Piezo1 leading to calcium influx using a similar experimental setup (Luo et al., 2022). Interestingly, it was recently identified that Piezo1 directly binds to E-cadherin, and Piezo1 is tethered to the actin cytoskeleton *via* the cadherin-β-catenin complex (Wang et al., 2022). While these findings help support the idea of cell–cell junction stabilization during mechanical perturbation, it remains enigmatic how compressive stress could increase recruitment of E-cadherin to the cell membrane. Surface expression of E-cadherin would be a balance of endocytosis and vesicular trafficking to the plasma membrane (Kowalczyk and Nanes, 2012). Imbalance of trafficking of E-cadherin, for instance, by reduced constitutive endocytosis, would alter the surface level of E-cadherin. We and others have shown that endocytosis is reduced when cell tension is elevated (Tan, Heureaux, and Liu, 2015; Willy et al., 2017; Wu et al., 2017). Thus, it is plausible that elevated tension, due to the application of compressive stress, could slow down the turnover of E-cadherin on the cell surface. It becomes more interesting considering that cancer cells are softer than normal cells (Lee and Liu, 2015; Alibert, Goud, and Manneville, 2017). A softer cell would become more deformed for a given compressive stress compared to a stiffer cell. In this way, the plasma membrane of cancer cells becomes more stretched by compressive stress, thereby resulting in reduced endocytosis and membrane protein turnover. Whether this could contribute to the increased E-cadherin level at the cell–cell junctions in cancer cells in response to compressive stress remains to be further investigated.

It will also be of great interest to understand how upregulation of cell–cell adhesion translates to cells detaching from the solid tumor and migrating to adjacent tissues (Mendonsa, Na, and Gumbiner, 2018). The fluid-like transition we observe occurs when mechanical compression strengthens adherens junctions, maintaining high cell density and enabling cancer cells to migrate collectively in a coordinated manner. Recent studies found that the deregulation of adherens junctions in epithelial migration results in a transition from coordinated to uncoordinated collective movement, whereas increased collagen density leads to local cell individualization (Ilina et al., 2020). We found that the front velocities of migrating 4T1 cell sheets increased with E-cadherin KD, consistent with what was reported by Ilina et al. However, the application of compressive stress to E-cadherin KD cells reduced front velocity, indicating that compressive stress activates mechanotransduction pathways to influence collective cell migration. In cancer cells, heterogeneities in cell–cell adhesion within a tumor may be exacerbated by ECM confinement, allowing individual cells and cell clusters to move separately from their neighbors. Further studies will be necessary to decipher how these heterogeneities impact collective migration and invasion. Furthermore, the sensitivity of different cell types to compressive stress may be related to the characteristics of the nucleus. The nucleus is mechanosensitive, influences cellular force generation (Grosser et al., 2021), and may be actively involved in unjamming transitions. During wound healing, nuclear tension has been reported to decrease with distance from the wound edge, indicating higher tension in cells near the wound edge than within the bulk cell sheet (Déjardin et al., 2020).

Our findings suggest a new physical picture of tumor development and cancer invasion, in which compressive stress inhibits the migratory activity of normal epithelial cells and permits cancer cells to migrate rapidly as a cohesive collective. The way 4T1 cells react to mechanical compression stimulates dense cell sheets to structurally rearrange in an unjamming transition that is not primarily driven by EMT. Cell–cell adhesion is an important regulator of jamming–unjamming transitions, and increased adhesion strength and attenuation of traction forces within the bulk cell sheet govern unjamming transitions under compression.

## Materials and methods

### Cell culture

The human non-tumorigenic breast epithelial cell line MCF10A was a gift from Sofia Merajver (University of Michigan) and was obtained from Dr. Heppner at the Michigan Cancer Foundation, where the cell line was originally developed. The mouse breast cancer cell line 4T1 was a gift from Lance Munn (Harvard Medical School) and was originally obtained from ATCC. The mouse breast cancer cell line 67NR was obtained from the Karmanos Cancer Institute (Detroit, MI). All cell lines were cultured in an RPMI medium (Corning) supplemented with 10% FBS, except for MCF10A. MCF10A cells were cultured in DMEM/F12 medium (Corning) supplemented with 5% horse serum, 20 ng/ml epidermal growth factor (EGF), 0.5 μg/ml hydrocortisone, 100 ng/ml cholera toxin, and 10 μg/ml insulin. Cells were cultured in a humidified atmosphere containing 5% CO_2_ at 37°C.

### Generation of E-cadherin knockdown 4T1 cells

4T1 cells expressing inducible shRNA knockdown of E-cadherin were generated using a transfer plasmid provided by Valerie Weaver at UCSF (Muncie et al., 2020). The transfer vector consisted of a modified pLKO.1 neo plasmid (Addgene) with the expression of the shRNA sequences under the control of 3× copies of the lac operator and a copy of the mNeonGreen fluorescent protein. The E-cadherin shRNA had the following sequence: 5’—GAA​CGA​GGC​TAA​CGT​CGT​AAT—3’; scramble shRNA (Sigma #SHC002) had the following sequence: CCG​GCA​ACA​AGA​TGA​AGA​GCA​CCA​ACT​CGA​GTT​GGT​GCT​CTT​CAT​CTT​GTT​GTT​TTT. Lentiviruses were generated by transfecting HEK 293T cells with the transfer vector, psPAX2 packaging vector, and pMD2. G envelope vector. Viral supernatant was collected 48 h after transfection. 4T1 cells were transduced with E-cadherin shRNA or scramble non-targeting control shRNA in RPMI medium, and after 24 h, shE-cadherin and scramble cells were selected with 200 μg/ml G-418 (Sigma) and 2 μg/ml puromycin, respectively. E-cadherin knockdown was induced in the shE-cadherin cells by adding 200 μM isopropyl-β-D-thiogalactoside (IPTG; Sigma) 72 h prior to experiments.

### Mechanical compression using an *in vitro* compression setup

Vertical compression was applied by adding a weight of 26 or 52 g over an area of 426 mm^2^ to achieve stresses of 600 or 1,200 Pa, respectively, using a previously established setup (Tse et al., 2012; Luo et al., 2022). The fixed weight applies constant stress to a UV-treated 1% agarose gel cushion in contact with cells. The agarose gel allows nutrient and oxygen diffusion. The agarose gel was used without the weight as a negative control. Samples for live imaging, immunofluorescence staining, and RT-qPCR were compressed for the specified durations under cell culture conditions.

### *In vitro* scratch-wound assay and time-lapse imaging

Cells were plated onto 6-well plates 72 h prior to experiments and grown to confluence. MCF10A and 4T1 cells were serum-starved in DMEM/F12 medium without horse serum and EGF and RPMI media without FBS, respectively, for 4 h prior to experiments. The cells were then incubated with Hoechst 33342 diluted in PBS for 30 min at 37°C. The cells were washed with PBS and placed in a complete medium. Scratches were created using a p-200 pipette tip to induce migration. After wounding, vertical compressive stresses of 0, 600, and 1,200 Pa were applied. Images of the wound area were captured using a ×4 objective at 30-min time points for 16 h using fluorescence microscopy on a Cytation 5 automated plate reader. Collective cell migration was quantified by measuring the area between wound edges using MATLAB. Spatiotemporal velocity maps were generated using the software AveMap+ (Deforet et al., 2012). The results were collected from three independent experiments.

### Immunofluorescence staining

Cells on glass coverslips were washed with PBS and fixed with 4% paraformaldehyde for 10 min, washed with PBS, and permeabilized with 0.1% Triton X-100 in PBS for 10 min. The cells were washed with PBS and blocked with 3% BSA in PBS for 1 h. The cells were incubated with a rabbit anti-E-cadherin antibody at 1:400 (Cell Signaling) and a primary mouse anti-vinculin antibody at 1:800 (Sigma–Aldrich) in 3% BSA overnight at 4°C. The cells were washed 3× with PBS and incubated with DAPI, phalloidin, and secondary antibodies in 3% BSA for 1 h at room temperature. The cells were washed 3× with PBS, mounted onto glass slides with Fluoromount-G (Invitrogen), and imaged by spinning disk confocal microscopy. Fluorescence levels relative to the control condition were quantified. E-cadherin at cell–cell contacts was quantified by measuring the fluorescence intensity at the cell membrane.

### Confocal microscopy

Images of immunostained cells were taken using an oil immersion Plan-Apochromat 60 x/1.4 NA objective on an inverted microscope (Olympus IX-81) equipped with an iXON3 EMCCD camera (Andor Technology), AOTF-controlled lasers (Andor Technology), and a Yokogawa CSU-X1 spinning disk confocal. Acquisition of images was controlled by MetaMorph (Molecular Devices). Single and z-stack images of cells fluorescently labeled for DAPI, F-actin (by 488-phalloidin), E-cadherin, and vinculin were captured with 405 nm, 488 nm, 561 nm, and 640 nm excitations, respectively, at exposure times of 200–500 ms.

### Quantification of cell and nuclear shapes

Immunofluorescence images were acquired as described above. For each pressure condition, more than 200 cells and nuclei were manually traced using ImageJ software (National Institutes of Health) from ten different fields of view. The cell and nuclear shape indices were computed for each traced cell and nucleus, respectively.

### Quantification of vinculin intensity

Cells were immunostained for E-cadherin using a rabbit anti-E-cadherin antibody (Cell Signaling) and vinculin using a primary mouse anti-vinculin antibody (Sigma–Aldrich), followed by fluorophore-conjugated secondary antibodies. ImageJ was used to threshold the image and compute the fluorescence intensity of particles at the basal plane. E-cadherin staining was used to define a mask at intercellular contacts, and vinculin fluorescence intensity was measured within the mask at the same plane as E-cadherin.

### RNA extraction and RT-qPCR

RNA was extracted using the RNeasy micro kit (Qiagen). RNA quality and quantity were measured using a NanoDrop 1000 spectrophotometer. Reverse transcription was performed using the iScript cDNA synthesis kit (Bio-Rad). qPCR assays were conducted using SYBR Green (Bio-Rad) and specific primers quantifying GAPDH, CDH1, CDH2, and VIM (OriGene) on the Bio-Rad CFX thermocycler. GAPDH was used as a control for quantifying relative gene expression. Mean C_t_ values from duplicates were used to calculate ΔC_t_ values normalized to GAPDH. Relative transcript levels were determined by calculating the change between ΔC_t_ values of the control and compressed samples as ΔΔC_t_ and calculating 2^−ΔΔCt^. The results were collected in duplicates from three independent experiments.

### Fabrication of soft silicone substrates

CY52-276 A/B (Dow Corning) with an A:B ratio of 1:1 was cast in 35 mm glass bottom dishes. After 10 min of degassing, the silicone substrates were cured on a hot plate at 70°C for 30 min. The substrates were then exposed to deep UV light for 5 min 19 mg EDC (1-Ethyl-3-(3-dimethylaminopropyl)-carbodiimide) (Thermo Fisher), 11 mg sulfo-NHS (N-Hydroxysulfosuccinimide) (Thermo Fisher), and 15 μl 2% w/v 0.5 μm carboxylate fluorescent beads (Thermo Fisher) were added to 1 ml DI water. The substrates were incubated with this suspension for 30 min to conjugate fluorescent beads to the surface of the soft silicone (Bashirzadeh et al., 2018a; Bashirzadeh et al., 2018b).

### Micropatterning of silicone substrates

Micropatterned substrates were made with the standard soft lithography technique to create a silicon master mold. Polydimethylsiloxane (PDMS) was prepared by mixing Sylgard-184 elastomer and curing agent (Dow Corning) in a 10:1 (w/w) ratio. After 10 min of degassing, the PDMS mixture was poured over the master and cured overnight at 60°C. PDMS stamps with 400 µm circular patterns were incubated with fibronectin (Corning) at a concentration of 40 μg/ml for 1 h. Soft silicone substrates were UV-treated for 5 min and then immediately placed in contact with the stamps (Tan et al., 2015). Printed substrates were passivated with an anti-adherence rinsing solution (STEMCELL Technologies) for 1 h.

### Traction force microscopy

A seeding density of 20,000 cells cm^−2^ formed confluent monolayers after 72 h. The same seeding density formed cell islands on micropatterned substrates after 48 h. Cells labeled by Hoechst 33342 and red fluorescence beads on the substrate surface were imaged before and after the removal of the cells using 10% sodium dodecyl sulfate (SDS). PIVlab (Thielicke and Stamhuis, 2014) was used to process image pairs (bead images before and after cell removal) and then used with the fast Fourier transform window deformation method to quantify the displacement of the beads, resulting in a displacement vector field. The Young’s modulus of the substrate was previously measured to be 7.2 kPa using sphere indentation (Bashirzadeh et al., 2019). Fourier transform traction cytometry was used to compute the traction stress field using MATLAB (Butler et al., 2002; Schwarz et al., 2002; Sabass et al., 2008; Plotnikov et al., 2014). To compute traction stresses at the leading edge during wound healing, regions parallel to the wound margin and within 5–7 cell layers were analyzed, and the rest of the cells were considered as bulk.

### Statistical analysis

Statistical analysis was carried out in Origin and performed with one-way ANOVA followed by Tukey post-hoc multiple comparisons test. Analysis of wound healing assays was performed by comparing the percentage of the wound closure at 16 h between different conditions. Results were collected from three independent experiments, and data from individual cells, the field of view, or cell islands are plotted as mean ± S.E. or shown as boxplots, depending on the experiment. Statistical significance was denoted by asterisks in the figure panels, with ∗ = *p* < 0.05, ∗∗ = *p* < 0.01, ∗∗∗ = *p* < 0.001.

### Theoretical model and simulation details

Cells in a 2D monolayer in the SPV model (Bi et al., 2016) are represented by polygons determined from a Voronoi tessellation of their center positions ( $r_{i}$ ). The center of each cell obeys the over-damped equation of motion

$$\frac{dr_{i}}{dt}=\mu F_{i}+v_{0}{\hat{n}}_{i}.$$ (1)

The first term on the right-hand side (RHS) of Eq. 1 comes from cell–cell interactions and the mechanical behavior of a single cell. Here, μ is the single-cell mobility constant, and the interaction force is given by $F_{i}=-\nabla_{i}E$ , where E is the total mechanical energy of the tissue given by

$$E=\overset{N}{\underset{i=1}{\sum }}\left[K_{A}{\left(A_{i}-A_{0}\right)}^{2}+K_{P}{\left(P_{i}-P_{0}\right)}^{2}\right].$$ (2)

In this equation, $A_{i}$ and $P_{i}$ are the area and perimeter of i th cell, and $A_{0}$ and $P_{0}$ are the cell preferred area and perimeter, respectively. $K_{A}$ and $K_{P}$ are the area and perimeter moduli. The term involving cell area models the cell’s incompressibility and monolayer’s resistance to height fluctuation. The quadratic term in the perimeter results from active contractility of the subcellular cortex. The linear term in the perimeter is a combination of the cortical tension and membrane tension due to cell–cell adhesion. The membrane line tension can be reduced by either increasing cell–cell adhesion, which encourages the cell to lengthen its shared edges with its neighbors, or by reducing actin–myosin contractility. Therefore, $P_{0}$ is positively correlated to cell–cell adhesion and negatively correlated to cell contractility (Farhadifar et al., 2007). For simplicity, we assume that the contribution of cell–cell adhesion to line tension is greater than the contribution of actin–myosin contractility, so $P_{0}$ is positive as a consequence. N is the total number of cells in the monolayer. To nondimensionalize cell shape quantities, we adapt a target shape index parameter $p_{0}=P_{0}/\sqrt{A_{0}}$ .

In addition to the interaction force, cells are driven by a self-propelled force due to their own polarized motility. In the SPV model, this is captured by the second term on the RHS of Eq. 1, where $v_{0}/\mu$ is the self-propulsive force magnitude. For each cell, this force acts along a polarization vector, given by

$${\hat{n}}_{i}=\left(\cos\theta_{i},\sin\theta_{i}\right).$$ (3)

This determines the direction of the self-propelled force. The polarity of cells is stochastic and obeys the rotational Brownian dynamics, given by

$$\frac{d\theta_{i}}{dt}=\eta_{i}\left(t\right),$$ (4)

where $\eta_{i}\left(t\right)$ is a white noise process with zero mean and variance 2 $D_{r}$ , given by

$$\langle \eta_{i}\left(t\right)\eta_{j}\left(t^{\prime }\right)\rangle =2D_{r}\delta \left(t-t^{\prime }\right)\delta_{ij}.$$ (5)

The magnitude of the rotational noise $D_{r}$ defines a time scale $\tau =1/D_{r}$ of persistent motion.

We employ an open-source implementation of the SPV model to perform CellGPU (Sussman, 2017) simulations of 900 cells for 5 million time steps with dt = 0.05 at each parameter of $p_{0}$ and $v_{0}$ shown in Figure 6. We choose $D_{r}=1,K_{A}=1,K_{P}=1,A_{0}=1$ .

In the SPV model, the traction force or the total force exerted by the cell onto the substrate is given by a sum of the viscous friction between the cell and the substrate and the self-propulsive force

$$f_{traction}=\frac{{-v}_{i}}{\mu }+\frac{v_{0}{\hat{n}}_{i}}{\mu }.$$ (6)

Since the net force on each cell is balanced according to Eq. 1, the traction $f_{traction}$ is always equivalent to the interaction force. Therefore, we use the interaction force to determine the magnitude of the traction of every cell in the system.

### Mapping condition of cells *in vitro* to theoretical simulation

To connect the experimental results to our theoretical model, we use 1) the observed cell aspect ratio (AR) values and 2) the cell traction forces to map the conditions of 4T1 and MCF10A cells to theoretical simulation parameters.

We performed simulations of the SPV model for a large range of $v_{0}$ – $p_{0}$ values. For each simulation step, we computed the cell AR of each cell using the open-source function. The mean aspect ratio for each simulation was then calculated by averaging the AR over every cell over simulation time steps. A contour map of mean AR in the $v_{0}$ – $p_{0}$ space is shown in Supplementary Figure S7. On the other hand, the experimental values of the mean AR for each condition are labeled in the $v_{0}$ – $p_{0}$ space and the locus of ( $v_{0},{\;p}_{0}$ ) pairs having these values of AR form contours. However, this alone does not give a definitive value of ( $v_{0}$ , $p_{0}$ ). The jamming phase boundary shown in Figure 6 is adapted from Bi et al. (2016) and demarcates the solid-like states from the fluid-like states, which is determined from the collective diffusive property at the tissue level. We next map the experimental values of cell tractions to the theoretical ones.

The average traction magnitude of the simulation systems that are on these contours is computed. We denoted the average traction magnitude to be T . To connect experimental traction values to the simulations, we choose the traction values of 4T1 cells in the compressed case to map to the simulation traction value at ( $p_{0}=3.404$ ; $v_{0}=0.71$ ). As a result, T (4T1 control) = 4.4790 was mapped to simulation traction of T ( $p_{0}=3.404$ ; $v_{0}=0.71$ ) = 0.626. This is an assumption in the analysis. However, the particular choice of this mapping does not influence the final qualitative conclusion in our work. The simulation traction magnitude of the system at this position is divided by the experimentally measured 4T1 traction to obtain the conversion factor. The traction values of other cell types and conditions are converted to simulation units by multiplying them by the factor of conversion. The positions of the other cell conditions on the phase diagram are then determined by matching both the AR and traction values between the experiment and the simulation.

## Data Availability

The raw data supporting the conclusions of this article will be made available by the authors, without undue reservation.
