# Supplementary material for: Compressive stress drives adhesion-dependent unjamming transitions in breast cancer cell migration
Source: Front Cell Dev Biol. 2022 Oct 4;10:933042. doi: 10.3389/fcell.2022.933042 (PMC9577106; doi:10.3389/fcell.2022.933042)
Supplement: Supplementary file 3 [file DataSheet1.PDF]

Compressive stress drives adhesion-dependent unjamming transitions in breast cancer cell migration

Grace Cai[1], Anh Nguyen[2], Yashar Bashirzadeh[3], Shan-Shan Lin[3], Dapeng Bi[2] and Allen P. Liu[1,3,4,5,6,*]

[1]Applied Physics Program, University of Michigan, Ann Arbor, MI, USA

[2]Department of Physics, Northeastern University, Boston, MA, USA

[3]Department of Mechanical Engineering, University of Michigan, Ann Arbor, MI, USA

[4]Department of Biomedical Engineering, University of Michigan, Ann Arbor, MI, USA

[5]Department of Biophysics, University of Michigan, Ann Arbor, MI, USA

[6]Cellular and Molecular Biology Program, University of Michigan, Ann Arbor, MI, USA

*** Correspondence:**

A.P.L.: allenliu@umich.edu

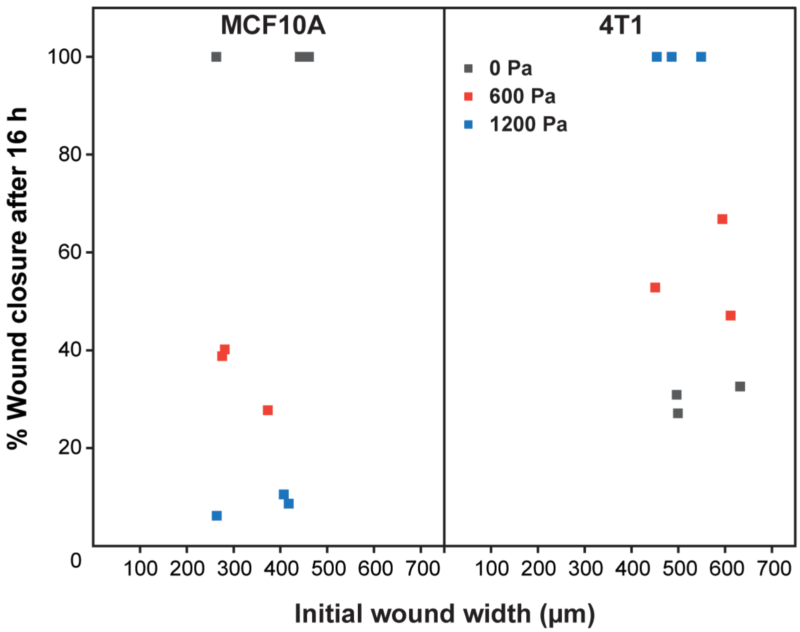

Figure S1. Percentage of wound closure after 16 h does not depend on initial wound width in wound healing assays for MCF10A and 4T1 cells under different compressive stresses.

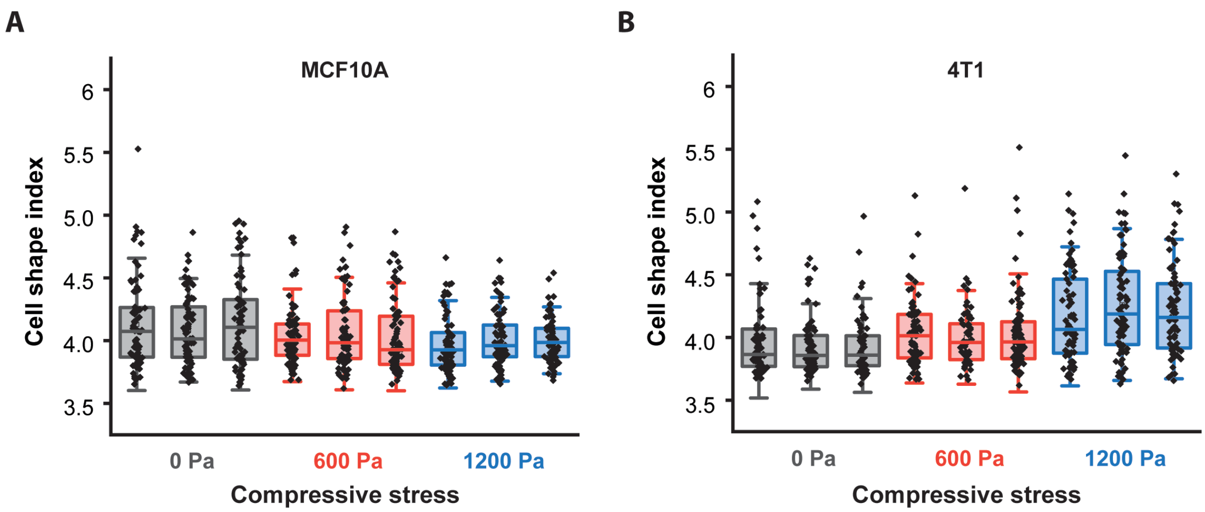

Figure S2. Cell shape index measurements of MCF10A **(A)** and 4T1 **(B)** under different compressive stresses are similar for independent experiments. Each boxplot shown displays mean ± S.D. for data from a single day of experiment.

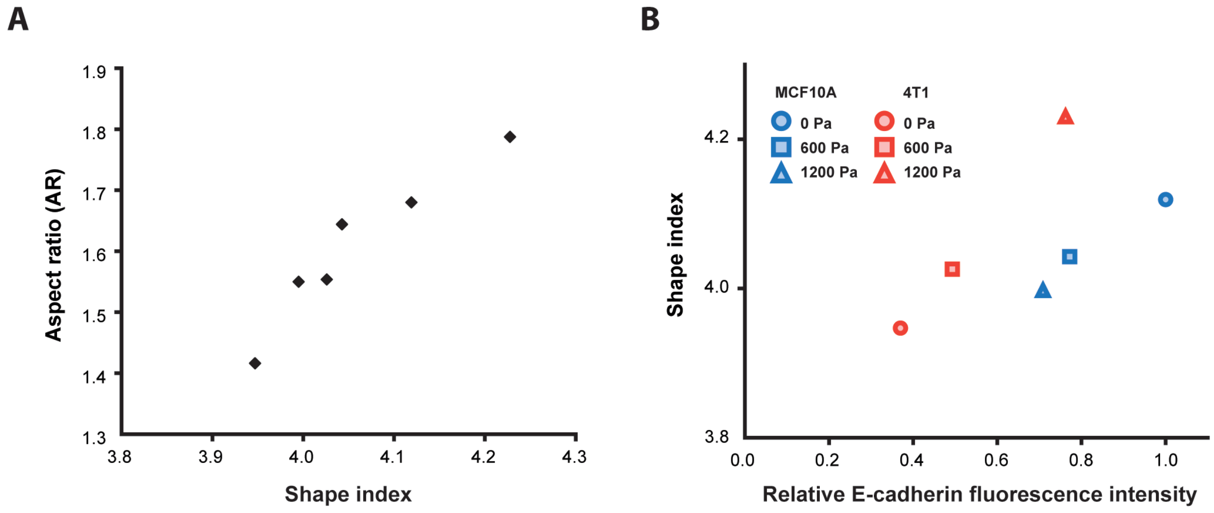

Figure S3. (A) Plot shows shape index, which emphasizes perimeter, vs. aspect ratio (AR), which emphasizes elongation. Substantial increases in AR are accompanied by smaller increases in shape index. **(B)** Plot shows E-cadherin fluorescence level vs. cell shape index for MCF10A and 4T1 under specified compressive stresses. As the relative E-cadherin fluorescence intensity at cell-cell contacts is elevated, the shape index increases as well.

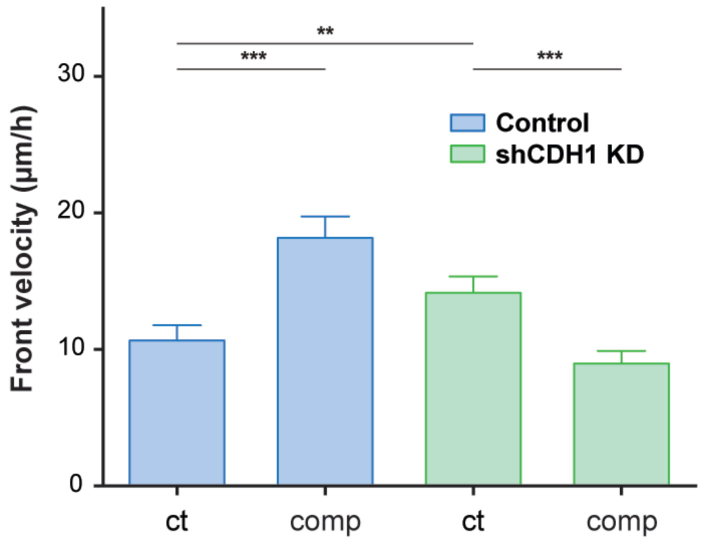

Figure S4. Front velocities were computed for migrating 4T1 cell sheets by averaging the velocity measurements within 228 μm from the leading edge over 16 h. Cell velocity increases when shCDH1 is KD (compared to control cells) and decreases upon compression (1200 Pa) in KD cells. Data represent n = 9 wound edges for each condition. Mean velocity ± S.E. is plotted from 3 independent experiments.

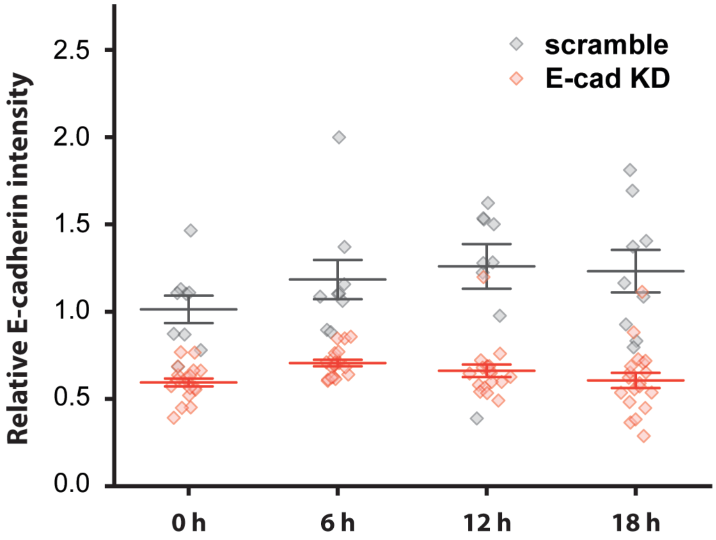

Figure S5. E-cad KD is stable over 18 hours as measured by immunofluorescence of E-cadherin at the cell membrane starting from 72 h following treatment with IPTG. Markers represent mean values ± S.E. of data from three independent experiments. Both scramble and E-cad KD data are normalized to the scramble condition at 0 h.

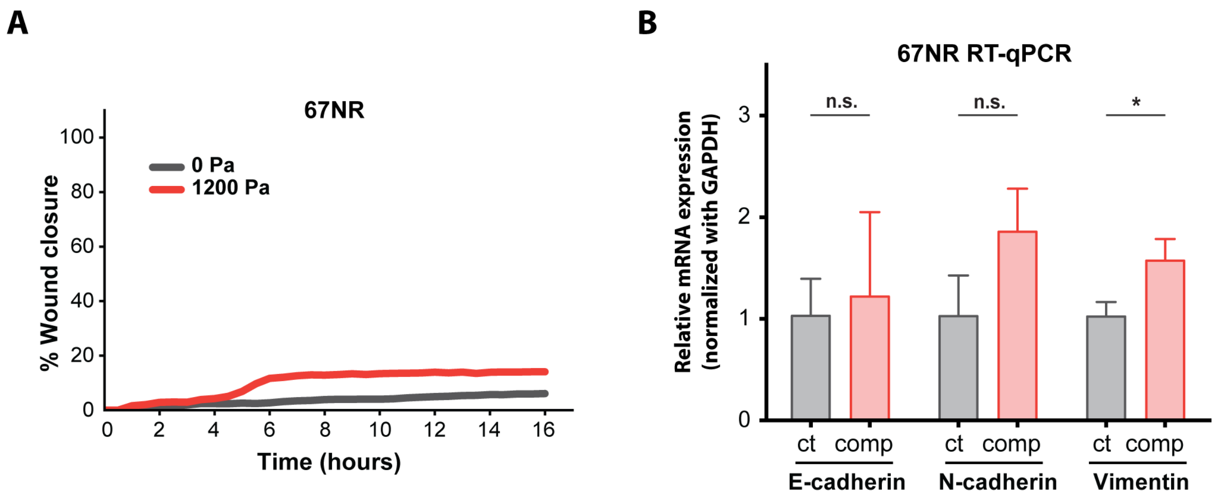

Figure S6. (A) Quantification of wound area for 67NR cells with and without compression. Mean wound area at each time point is plotted from 3 independent replicates as a representative trace. **(B)** qPCR analysis of E-cadherin, N-cadherin, and vimentin mRNA levels with and without compression (1,200 Pa). Transcript levels are calculated using the $\Delta\Delta C_t$ method normalized to GAPDH. Mean mRNA level ± S.E. is plotted from 3 independent experiments with duplicates per experiment.

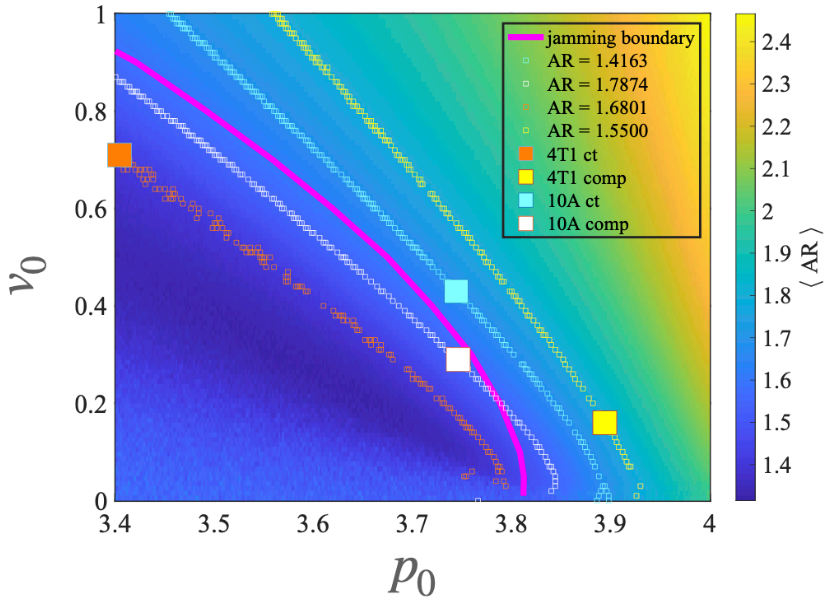

Figure S7. Contour plot of mean AR at different v_0 and p_0 is represented as a color plot. The empty squares represent contour lines with the particular value of AR observed in experiments. The filled squares represent different cell lines and experimental conditions mapped by extrapolating from cell traction.

Description of Additional Supplementary Files

Supplementary Movie 1. Collective cell migration of control MCF10A cells during a scratch wound assay. A confluent MCF10A monolayer is scratched and the wound edge is tracked by time lapse fluorescence imaging. Cell nuclei are labeled with Hoechst 33342.

Supplementary Movie 2. Collective cell migration of control 4T1 cells during a scratch wound assay. A confluent 4T1 monolayer is scratched and the wound edge is tracked by time lapse fluorescence imaging. Cell nuclei are labeled with Hoechst 33342.

Supplementary Movie 3. Collective cell migration of compressed MCF10A cells during a scratch wound assay. A confluent MCF10A monolayer is scratched and compressed by 1,200 Pa. The wound edge is tracked by time lapse fluorescence imaging. Cell nuclei are labeled with Hoechst 33342.

Supplementary Movie 4. Collective cell migration of compressed 4T1 cells during a scratch wound assay. A confluent 4T1 monolayer is scratched and compressed by 1,200 Pa. The wound edge is tracked by time lapse fluorescence imaging. Cell nuclei are labeled with Hoechst 33342.
